# Morphology and multigene phylogeny reveal a new order and a new species of wood-inhabiting basidiomycete fungi (Agaricomycetes)

**DOI:** 10.3389/fmicb.2022.970731

**Published:** 2022-08-31

**Authors:** Kai-Yue Luo, Chang-Lin Zhao

**Affiliations:** ^1^Key Laboratory for Forest Resources Conservation and Utilization in the Southwest Mountains of China, Ministry of Education, Southwest Forestry University, Kunming, China; ^2^Yunnan Key Laboratory of Plateau Wetland Conservation, Restoration and Ecological Services, Southwest Forestry University, Kunming, China; ^3^College of Biodiversity Conservation, Southwest Forestry University, Kunming, China; ^4^Yunnan Key Laboratory for Fungal Diversity and Green Development, Kunming Institute of Botany, Chinese Academy of Science, Kunming, China

**Keywords:** biodiversity, fungal systematics, ITS, LSU, new taxa, wood-decaying fungi, Xenasmatales, *Xenasmatella nigroidea*

## Abstract

Dead wood-associated fungi play an important role in wood degradation and the recycling of organic matter in the forest ecological system. Xenasmataceae is a cosmopolitan group of wood-rotting fungi that grows on tropical, subtropical, temperate, and boreal vegetation. In this study, a new fungal order, Xenasmatales, is introduced based on both morphology and multigene phylogeny to accommodate Xenasmataceae. According to the internal transcribed spacer and nuclear large subunit (ITS+nLSU) and nLSU-only analyses of 13 orders, Xenasmatales formed a single lineage and then grouped with orders Atheliales, Boletales, and Hymenochaetales. The ITS dataset revealed that the new taxon *Xenasmatella nigroidea* clustered into *Xenasmatella* and was closely grouped with *Xenasmatella vaga*. In the present study, *Xenasmatella nigroidea* collected from Southern China is proposed as a new taxon, based on a combination of morphology and phylogeny. Additionally, a key to the *Xenasmatella* worldwide is provided.

## Introduction

Among eukaryotic microorganisms, wood-decaying fungi interact positively with dead wood, playing a fundamental ecological role as decomposers of plants in the fungal tree of life (James et al., 2020). Wood-associated fungi are cosmopolitan and rich in diversity since they grow on tropical, subtropical, temperate, and boreal vegetation (Gilbertson and Ryvarden, 1987; Núñez and Ryvarden, 2001; Bernicchia and Gorjón, 2010; Dai, 2012; Ryvarden and Melo, 2014; Dai et al., 2015, 2021; Wu et al., 2020).

Xenasmataceae Oberw., a typical wood-associated fungal group mainly distributed in the tropics was discovered by Oberwinkler (1966), and typified by *Xenasma* Donk. Three genera, namely, *Xenasma, Xenasmatella* Oberw., and *Xenosperma* Oberw., have been accommodated in this family, however, higher-level classification of the order has not been designated. The tenth edition of the Dictionary of the Fungi showed that Xenasmataceae belongs to Polyporales Gäum., and consists of three genera (Kirk et al., 2008). MycoBank indicates that Xenasmataceae has a higher classification within Polyporales, although the Index Fungorum shows that Xenasmataceae belongs to the order Russulales.

High phylogenetic diversity among corticioid homobasidiomycetes suggests a close relationship among Radulomyces M.P. Christ., *Xenasmatella*, and *Coronicium* J. Erikss. and Ryvarden. *Xenasma pseudotsugae* (Burt) J. Erikss. nested into the euagarics clade, in which it grouped with *Coronicium* and *Radulomyces*. The three taxa of *Radulomyces* grouped together with *Phlebiella pseudotsugae* (Burt) K.H. Larss. and Hjortstam and *Coronicium alboglaucum* (Bourdot and Galzin) Jülich, and were composed of a rather confusing group with no obvious morphological features or ecological specialization to tie these three genera together (Larsson et al., 2004). The classification of corticioid fungi with 50 putative families from published preliminary analyses and phylogenies of sequence data showed that three species of *Xenasmatella* formed a single lineage with strong support within the unplaced Phlebiella family, in which this clade was unclaimed to any orders (Larsson, 2007). A higher-level phylogenetic classification of the Kingdom Fungi revealed that the Phlebiella clade and Jaapia clade do not show affinities within any orders (Hibbett et al., 2007). An outline of all genera of Basidiomycota with combined SSU, ITS, LSU, tef1, rpb1, and rpb2 datasets showed that *Xenasmatella* was assigned to Xenasmataceae within the order Russulales (He et al., 2019). Therefore, there is debate on the classification at the order level for the Xenasmataceae.

Recently, *Xenasmatella* has been studied deeply on the basis of morphology and phylogeny. *Phlebiella* P. Karst. was deemed to have not been legitimately published previously, and the name *Xenasmatella* was accepted (Duhem, 2010; Larsson et al., 2020; Maekawa, 2021). Molecular systematics involving *Xenasmatella* was carried out recently. On the basis of morphological and molecular identification, Zong et al. (2021) studied the sequences of 27 fungal specimens representing 24 species between the *Xenasmatella* clade and related orders; and the *Xenasmatella* clade formed a single lineage and three new species, namely, *X. rhizomorpha* C.L. Zhao, *X. tenuis* C.L. Zhao, and *X. xinpingensis* C.L. Zhao. Both the MycoBank database (http://www.MycoBank.org) and Index Fungorum (http://www.indexfungorum.org, accessed on June 20, 2022) have recorded 41 specific and infraspecific names in *Xenasmatella*. To date, the number of *Xenasmatella* species accepted worldwide has reached 25 (Oberwinkler, 1966; Stalpers, 1996; Hjortstam and Ryvarden, 2005; Bernicchia and Gorjón, 2010; Duhem, 2010; Larsson et al., 2020; Maekawa, 2021), of which, nine species have been found in China (Dai et al., 2004; Dai, 2011; Huang et al., 2019; Zong and Zhao, 2021; Zong et al., 2021).

In the present study, we verified the taxonomy and phylogeny of Xenasmataceae. In addition, we analyzed the species diversity of Xenasmataceae and constructed a phylogeny to the order level of this family on the basis of large subunit nuclear ribosomal RNA gene (nLSU) sequences, the internal transcribed spacer (ITS) regions, and ITS+nLSU analyses. Based on both morphology and phylogeny, we propose a new fungal order, Xenasmatales and a new species, *Xenasmatella nigroidea*. A key to the 25 accepted species of *Xenasmatella* worldwide is also provided.

### The accepted species list

*Xenasma* Donk (1957).

1. *Xenasma Aculeatum* C.E. Gómez (1972).
2. *Xenasma Amylosporum* Parmasto (1968).
3. *Xenasma Longicystidiatum* Boidin and Gilles (2000).
4. Xenasma Parvisporum Pouzar (1982).
5. *Xenasma Praeteritum* (H.S. Jacks.) Donk (1957).
6. *Xenasma Pruinosum* (Pat.) Donk (1957).
7. *Xenasma Pulverulentum* (H.S. Jacks.) Donk (1957).
8. *Xenasma Rimicola* (P. Karst.) Donk (1957).
9. *Xenasma Subclematidis* S.S. Rattan (1977).
10. *Xenasma Tulasnelloideum* (Höhn. and Litsch.) Donk (1957).
11. Xenasma Vassilievae Parmasto (1965).

*Xenasmatella* Oberwinkler (1966).

1. *Xenasmatella Ailaoshanensis* C.L. Zhao ex C.L. Zhao and T.K. Zong (2021).
2. *Xenasmatella Alnicola* (Bourdot and Galzin) K.H. Larss. and Ryvarden (2020).
3. *Xenasmatella Ardosiaca* (Bourdot and Galzin) Stalpers (1996).
4. *Xenasmatella Athelioidea* (N. Maek.) N. Maek. (2021).
5. *Xenasmatella Bicornis* (Boidin and Gilles) Piatek (2005).
6. *Xenasmatella Borealis* (K.H. Larss. and Hjortstam) Duhem (2010).
7. *Xenasmatella Caricis-Pendulae* (P. Roberts) Duhem (2010).
8. *Xenasmatella Christiansenii* (Parmasto) Stalpers (1996).
9. *Xenasmatella Cinnamomea* (Burds. and Nakasone) Stalpers (1996).
10. *Xenasmatella Fibrillosa* (Hallenb.) Stalpers (1996).
11. *Xenasmatella Globigera* (Hjortstam and Ryvarden) Duhem (2010).
12. *Xenasmatella Gossypina* (C.L. Zhao) G. Gruhn and Trichies (2021).
13. *Xenasmatella Inopinata* (H.S. Jacks.) Hjortstam and Ryvarden (1979).
14. *Xenasmatella Insperata* (H.S. Jacks.) Jülich (1979).
15. *Xenasmatella Nasti* Boidin and Gilles ex Stalpers (1996).
16. *Xenasmatella Odontioidea* Ryvarden and Liberta (1978).
17. *Xenasmatella Palmicola* (Hjortstam and Ryvarden) Duhem (2010).
18. Xenasmatella Rhizomorpha C.L. Zhao (2021).
19. *Xenasmatella Romellii* Hjortstam (1983).
20. Xenasmatella Sanguinescens Svrček (1973).
21. *Xenasmatella Subflavidogrisea* (Litsch.) Oberw. ex Jülich (1979).
22. *Xenasmatella Tenuis* C.L. Zhao (2021).
23. *Xenasmatella Vaga* (Fr.) Stalpers (1996).
24. *Xenasmatella Wuliangshanensis* (C.L. Zhao) G. Gruhn and Trichies (2021).
25. Xenasmatella Xinpingensis C.L. Zhao (2021).

*Xenosperma* Oberw. (1966).

1. *Xenosperma Hexagonosporum* Boidin and Gilles (1989).
2. *Xenosperma Ludibundum* (D.P. Rogers and Liberta) Oberw. ex Jülich (1979).
3. *Xenosperma Murrillii* Gilb. and M. Blackw. (1987).
4. *Xenosperma Pravum* Boidin and Gilles (1989).

## Materials and methods

### Sample collection and herbarium specimen preparation

Fresh fruit bodies of fungi growing on the stumps of angiosperms were collected from Honghe, Yunnan Province, P.R. China. The samples were photographed *in situ*, and macroscopic details were recorded. Field photographs were taken by a Jianeng 80D camera. All photographs were focus stacked and merged using Helicon Focus software. Once the macroscopic details were recorded, the specimens were transported to a field station where they were dried on an electronic food dryer at 45°C. Once dried, the specimens were labeled and sealed in envelopes and plastic bags. The dried specimens were deposited in the herbarium of the Southwest Forestry University (SWFC), Kunming, Yunnan Province, P.R. China.

### Morphology

The macromorphological descriptions were based on field notes and photos captured in the field and laboratory. The color, texture, taste, and odor of fruit bodies were mostly based on the authors' field trip investigations. Rayner (1970) and Petersen (1996) were used for the color terms. All materials were examined under a Nikon 80i microscope. Drawings were made with the aid of a drawing tube. The measurements and drawings were made from slide preparations stained with cotton blue (0.1 mg aniline blue dissolved in 60 g pure lactic acid), melzer's reagent (1.5 g potassium iodide, 0.5 g crystalline iodine, 22 g chloral hydrate, and aq. dest. 20 ml), and 5% potassium hydroxide. Spores were measured from the sections of the tubes; and when presenting spore size data, 5% of the measurements excluded from each end of the range are shown in parentheses (Wu et al., 2022). The following abbreviations were used: KOH = 5% potassium hydroxide water solution, CB = cotton clue, CB– = acyanophilous, IKI = Melzer's reagent, IKI– = both inamyloid and indextrinoid, L = means spore length (arithmetic average for all spores), W = means spore width (arithmetic average for all spores), Q = variation in the L/W ratios between the specimens studied, and n = a/b (number of spores (a) measured from given number (b) of specimens).

### Molecular phylogeny

The CTAB rapid plant genome extraction kit-DN14 (Aidlab Biotechnologies Co., Ltd., Beijing, P.R. China) was used to obtain genomic DNA from the dried specimens following the manufacturer's instructions (Zhao and Wu, 2017). The nuclear ribosomal ITS region was amplified with the primers ITS5 and ITS4 (White et al., 1990). The nuclear nLSU region was amplified with the primer pairs LR0R and LR7 (http://lutzonilab.org/nuclear-ribosomal-dna/, accessed on September 12, 2021). The PCR procedure used for ITS was as follows: initial denaturation at 95°C for 3 min, followed by 35 cycles at 94°C for 40 s, 58°C for 45 s, and 72°C for 1 min, and a final extension of 72°C for 10 min. The PCR procedure used for nLSU was as follows: initial denaturation at 94°C for 1 min, followed by 35 cycles at 94°C for 30 s, 48°C for 1 min, and 72°C for 1.5 min, and a final extension of 72°C for 10 min. The PCR products were purified and sequenced at Kunming Tsingke Biological Technology Limited Company (Yunnan Province, P.R. China). All the newly generated sequences were deposited in the National Center for Biotechnology Information (NCBI) GenBank (https://www.ncbi.nlm.nih.gov/genbank/, accessed on September 12, 2021) (Table 1).

**Table 1:** The list of species, specimens, and GenBank accession numbers of sequences used in this study.

**Species Name**	**Specimen No**.	**GenBank Accession No**.		**References**
		**ITS**	**nLSU**	
*Albatrellus confluens*	PV 10193	–	AF506393	Larsson et al., 2004
*Aleurobotrys botryosus*	CBS 336.66	MH858812	MH870451	Vu et al., 2019
*Amaurodon viridis*	TAA 149664	AY463374	AY586625	Larsson et al., 2004
*Amphinema byssoides*	EL 1198	–	AY586626	Larsson et al., 2004
*Amylostereum areolatum*	NH 8041	–	AF506405	Larsson and Larsson, 2003
*Aphanobasidium pseudotsugae*	NH 10396	–	AY586696	Larsson et al., 2004
*Auriscalpium vulgare*	EL 3395	–	AF506375	Larsson and Larsson, 2003
*Athelia epiphylla*	EL 1298	AY463382	AY586633	Larsson et al., 2004
*Athelopsis subinconspicua*	KHL 8490	AY463383	AY586634	Larsson et al., 2004
*Bondarzewia dickinsii*	Li 150909/19	KX263721	KX263723	Unpublished
*Candelabrochaete septocystidia*	AS 95	–	EU118609	Larsson, 2007
*Chaetodermella luna*	NH 8482	EU118615	–	Larsson, 2007
*C. luna*	CBS 305.65	–	MH870216	Vu et al., 2019
*Chondrostereum purpureum*	EL 5997	–	AY586644	Larsson et al., 2004
*Clavulicium delectabile*	KHL 11147	–	AY586688	Larsson et al., 2004
*Clavulina cristata*	EL 9597	AY463398	AY586648	Larsson et al., 2004
*Columnocystis abietina*	KHL 12474	EU118619	–	Larsson, 2007
*Coronicium alboglaucum*	NH 4208	–	AY586650	Larsson et al., 2004
*Cystostereum murrayi*	KHL 12496	EU118623	–	Larsson, 2007
*Dacrymyces stillatus*	CBS 195.48	MH856306	MH867857	Vu et al., 2019
*Dacryopinax spathularia*	Miettinen 20559	MW191976	MW159092	Unpublished
*Erythricium laetum*	NH 14530	AY463407	AY586655	Larsson et al., 2004
*Exidia recisa*	SL Lindberg 180317	–	MT664783	Unpublished
*Exidiopsis calcea*	KHL 11075	–	AY586654	Larsson et al., 2004
*Gloeocystidiellum porosum*	FCUG 1933	–	AF310094	Larsson and Hallenberg, 2001
*Haplotrichum conspersum*	KHL 11063	AY463409	AY586657	Larsson et al., 2004
*Hydnocristella himantia*	KUC 20131001-35	–	KJ668382	Unpublished
*Hydnomerulius pinastri*	412	–	AF352044	Jarosch and Besl, 2001
*Hydnum repandum*	420526MF0827	–	MG712372	Unpublished
*Hygrophoropsis aurantiaca*	EL 4299	–	AY586659	Larsson et al., 2004
*Hymenochaete cinnamomea*	EL 699	AY463416	AY586664	Larsson et al., 2004
*Hyphodermella corrugate*	KHL 3663	–	EU118630	Larsson, 2007
*Hyphodontia aspera*	KHL 8530	AY463427	AY586675	Larsson et al., 2004
*Inonotus radiatus*	TW 704	–	AF311018	Wagner and Fischer, 2001
*Junghuhnia nitida*	CBS 45950	–	MH868226	Vu et al., 2019
*Kavinia alboviridis*	EL 1698	–	AY463434	Larsson et al., 2004
*Kavinia himantia*	LL 98	AY463435	AY586682	Larsson et al., 2004
*Lactarius volemus*	KHL 8267	–	AF506414	Larsson and Larsson, 2003
*Laetisaria fuciformis*	CBS 18249	–	MH868023	Vu et al., 2019
*Lentaria dendroidea*	SJ 98012	EU118640	EU118641	Larsson, 2007
*Lignosus hainanensis*	Dai 10670	NR154112	GU580886	Cui et al., 2011
*Merulicium fusisporum*	Hjm s.n.	EU118647	–	Larsson, 2007
*Mycoaciella bispora*	EL 1399	–	AY586692	Larsson et al., 2004
*Peniophora pini*	Hjm 18143	–	EU118651	Larsson, 2007
*Phanerochaete sordida*	KHL 12054	–	EU118653	Larsson, 2007
*Phellinus chrysoloma*	TN 4008	–	AF311026	Wagner and Fischer, 2001
*Phlebia nitidula*	Nystroem 020830	–	EU118655	Larsson, 2007
*Podoscypha multizonata*	CBS 66384	–	MH873501	Vu et al., 2019
*Polyporus tubiformis*	WD 1839	AB587634	AB368101	Sotome et al., 2011
*Porpomyces mucidus*	KHL 11062	AF347091	–	Unpublished
*P. mucidus*	Dai 10726	–	KT157839	Wu et al., 2015
*Pseudomerulius aureus*	BN 99	–	AY586701	Larsson et al., 2004
*Punctularia strigosozonata*	LR 40885	AY463456	AY586702	Larsson et al., 2004
*Rickenella fibula*	AD 86033	–	AY586710	Larsson et al., 2004
*Russula violacea*	SJ 93009	AF506465	AF506465	Larsson and Larsson, 2003
*Scopuloides hydnoides*	WEI 17569	–	MZ637283	Chen et al., 2021
*Sistotrema alboluteum*	TAA 167982	AY463467	AY586713	Larsson et al., 2004
*Sistotremastrum niveocremeum*	MAFungi 12915	–	JX310442	Telleria et al., 2013
*Sistotremastrum suecicum*	KHL 11849	–	EU118667	Larsson, 2007
*Sphaerobasidium minutum*	KHL 11714	–	DQ873653	Larsson et al., 2006
*Stereum hirsutum*	NH 7960	AF506479	–	Larsson and Larsson, 2003
*Tomentellopsis echinospora*	KHL 8459	AY463472	AY586718	Larsson et al., 2004
*Trametes suaveolens*	CBS 279.28	MH855012	MH866480	Vu et al., 2019
*Trechispora farinacea*	KHL 8793	AF347089	–	Larsson et al., 2004
*T. farinacea*	MAFungi 79474	–	JX392856	Telleria et al., 2013
*Tubulicrinis subulatus*	KHL 11079	AY463478	AY586722	Larsson et al., 2004
*Veluticeps abietina*	HHB 13663	–	KJ141191	Unpublished
*Veluticeps berkeleyi*	HHB 8594	–	HM536081	Garcia-Sandoval et al., 2010
*Vuilleminia comedens*	EL 199	AY463482	AY586725	Larsson et al., 2004
*Wrightoporia lenta*	KN 150311	–	AF506489	Larsson and Larsson, 2003
*Xerocomus chrysenteron*	EL 3999	AF347103	–	Larsson et al., 2004
*Xenasma praeteritum*	ACD 0185	OM009268		Unpublished
*Xenasma pruinosum*	OTU 1299	MT594801		Unpublished
*Xenasma rimicola*	NLB 1571	MT571671		Unpublished
*X*. *rimicola*	NLB 1449	MT537020		Unpublished
*Xenasmatella ailaoshanensis*	CLZhao 3895	MN487105	–	Huang et al., 2019
*X. ailaoshanensis*	CLZhao 4839	MN487106	–	Huang et al., 2019
*Xenasmatella ardosiaca*	CBS 126045	MH864060	MH875515	Vu et al., 2019
*Xenasmatella borealis*	UC 2022974	KP814210	–	Rosenthal et al., 2017
*X. borealis*	UC 2023132	KP814274	–	Rosenthal et al., 2017
*Xenasmatella christiansenii*	TASM YGG 26	MT526341	–	Gafforov et al., 2020
*X. christiansenii*	TASM YGG 36	MT526342	–	Gafforov et al., 2020
*Xenasmatella gossypina*	CLZhao 4149	MW545958	–	Zong and Zhao, 2021
*X. gossypina*	CLZhao 8233	MW545957	–	Zong and Zhao, 2021
*Xenasmatella nigroidea*	CLZhao 18300	OK045679	OK045677	Present study
*X. nigroidea*	CLZhao 18333 ^*^	OK045680	OK045678	Present study
*Xenasmatella rhizomorpha*	CLZhao 9156	MT832954	–	Zong et al., 2021
*X. rhizomorpha*	CLZhao 9847	MT832953	–	Zong et al., 2021
*Xenasmatella tenuis*	CLZhao 4528	MT832960	–	Zong et al., 2021
*X. tenuis*	CLZhao 11258	MT832959	–	Zong et al., 2021
*Xenasmatella vaga*	KHL 11065	EU118660	EU118661	Larsson, 2007
*X. vaga*	BHI-F 160a	MF161185	–	Haelewaters et al., 2018
*Xenasmatella wuliangshanensis*	CLZhao 4080	MW545962	–	Zong and Zhao, 2021
*X. wuliangshanensis*	CLZhao 4308	MW545963	–	Zong and Zhao, 2021
*Xenasmatella xinpingensis*	CLZhao 2216	MT832961	–	Zong et al., 2021
*X. xinpingensis*	CLZhao 2467	MT832962	–	Zong et al., 2021

* Indicates type materials.

**Figure 1:**
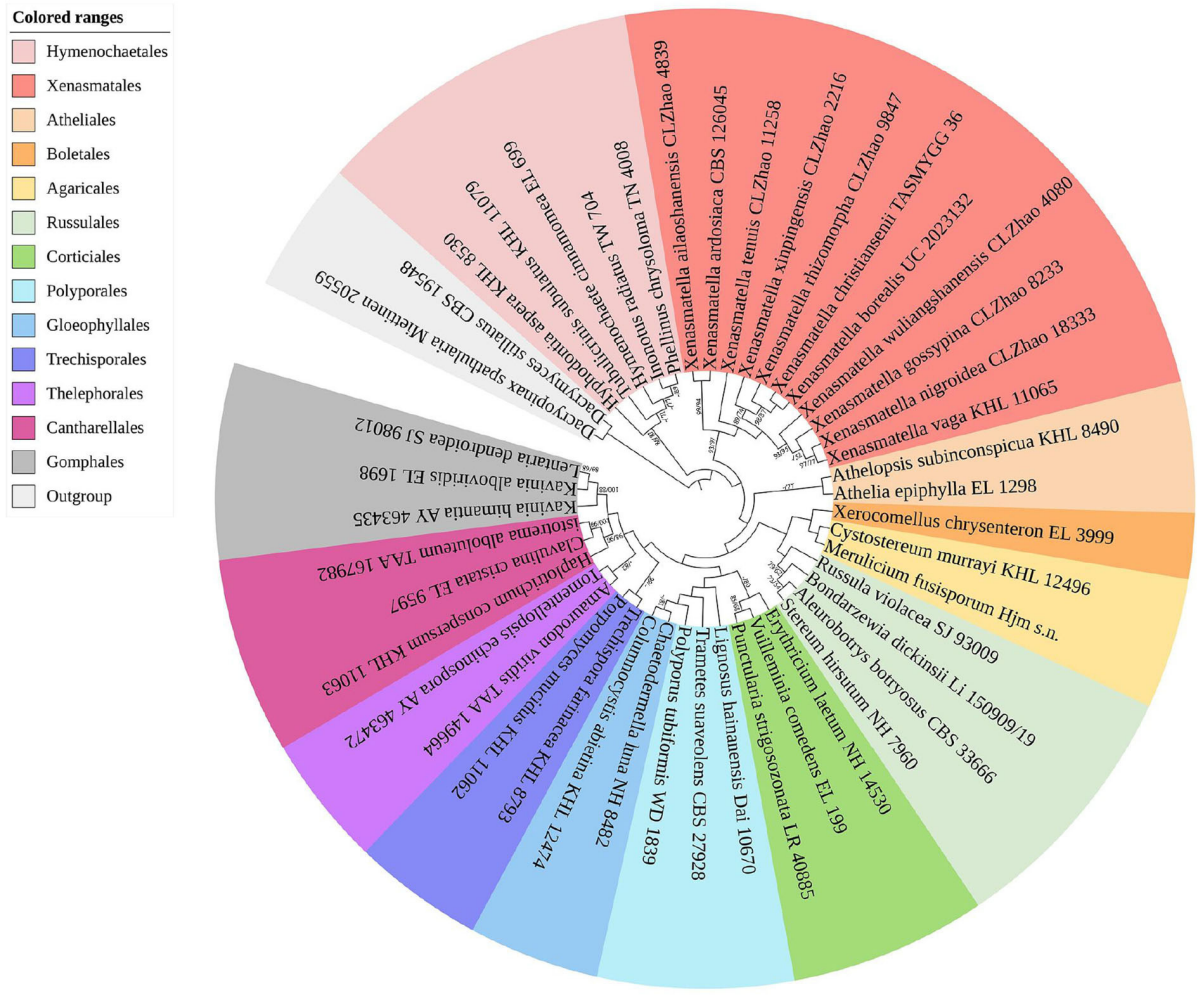
A maximum parsimony strict consensus tree illustrating the phylogeny of the new order Xenasmatales and related order in the class Agaricomycetes based on ITS+nLSU sequences. The orders represented by each color are indicated in the upper left of the phylogenetic tree. Branches are labeled with a maximum likelihood bootstrap value ≥ 70%, and a parsimony bootstrap value ≥ 50, respectively.

**Figure 2:**
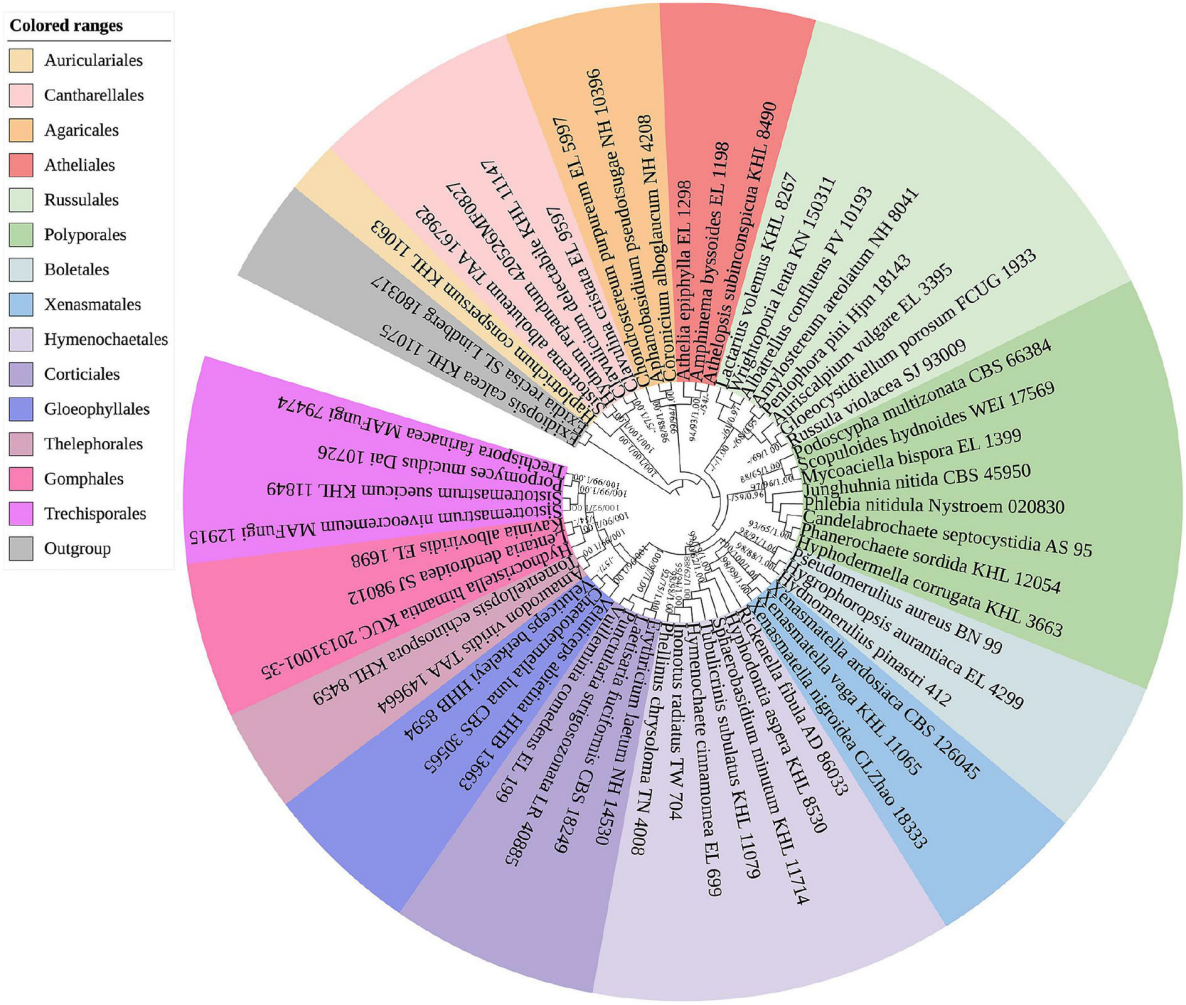
A maximum parsimony strict consensus tree illustrating the phylogeny of the new order Xenasmatales and related order in the class Agaricomycetes based on nLSU sequences. The orders represented by each color are indicated in the upper left of the phylogenetic tree. Branches are labeled with a maximum likelihood bootstrap value ≥ 70%, a parsimony bootstrap value ≥ 50%, and Bayesian posterior probabilities ≥ 0.95, respectively.

**Figure 3:**
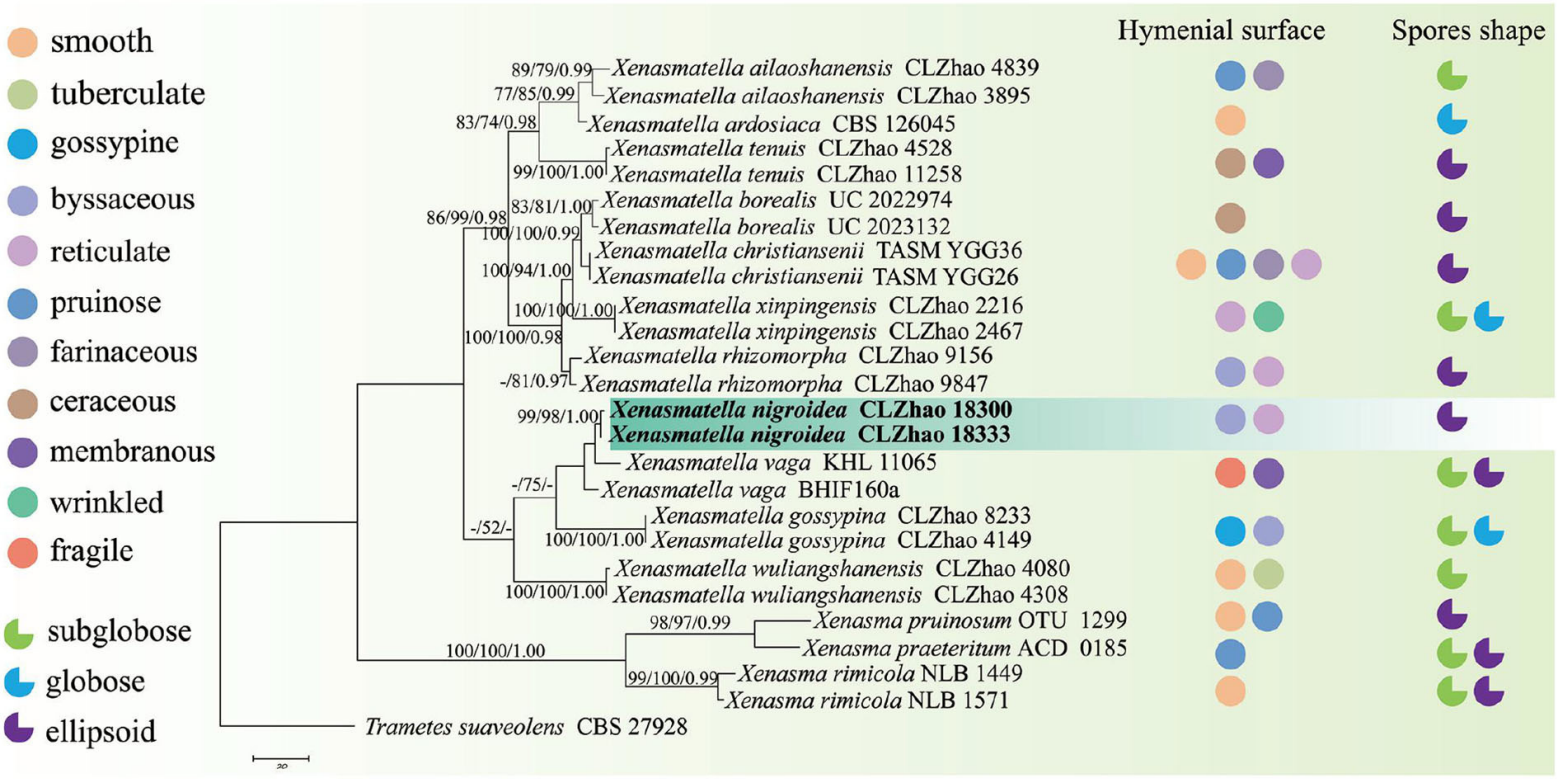
A maximum parsimony strict consensus tree illustrating the phylogeny of a new species and related species in *Xenasmatella* and *Xenasma* based on ITS sequences. Branches are labeled with a maximum likelihood bootstrap value ≥ 70%, a parsimony bootstrap value ≥ 50%, and Bayesian posterior probabilities ≥ 0.95, respectively. The new species are in bold.

The sequences and alignment were adjusted manually using AliView version 1.27 (Larsson, 2014). The datasets were aligned with Mesquite version 3.51. The ITS+nLSU dataset and the nLSU-only sequence dataset were used to position a new order, Xenasmatales, and the ITS-only dataset was used to position a new species among the *Xenasmatella*-related taxa. Sequences of *Dacrymyces stillatus* and *Dacryopinax spathularia* retrieved from GenBank were used as the outgroup for the ITS+nLSU sequences (Figure 1) (He et al., 2019); sequences of *Exidia recisa* and *Exidiopsis calcea* retrieved from GenBank were used as the outgroup for the nLSU sequences (Figure 2) (Larsson, 2007); and the sequence of *Trametes suaveolens* was used as the outgroup for the ITS-only sequences (Figure 3) (Zong and Zhao, 2021).

The three combined datasets were analyzed using maximum parsimony (MP), maximum likelihood (ML), and Bayesian inference (BI), according to Zhao and Wu (2017), and the tree was constructed using PAUP^*^ version 4.0b10 (Swofford, 2002). All characters were equally weighted and gaps were treated as missing data. Trees were inferred using the heuristic search option with TBR branch swapping and 1,000 random sequence additions. Max-trees were set to 5,000, branches of zero length were collapsed, and all parsimonious trees were saved. Clade robustness was assessed using the bootstrap (BT) analysis with 1,000 replicates (Felsenstein, 1985). Descriptive tree statistics—tree length (TL), consistency index (CI), retention index (RI), rescaled consistency index (RC), and homoplasy index (HI)—were calculated for each maximum parsimonious tree generated. In addition, multiple sequence alignment was analyzed using ML in RAxML-HPC2 through the Cipres Science Gateway (Miller et al., 2012). Branch support (BS) for ML analysis was determined by 1,000 bootstrap replicates.

MrModeltest 2.3 (Nylander, 2004) was used to determine the best-fit evolution model for each dataset of BI, which was performed using MrBayes 3.2.7a with a GTR+I+G model of DNA substitution and a gamma distribution rate variation across sites (Ronquist et al., 2012). A total of 4 Markov chains were run for 2 runs from random starting trees for 1 million generations for the ITS+nLSU dataset (Figure 1), 1.4 million generations for the nLSU-only sequences (Figure 2), and 0.5 million generations for the ITS-only sequences (Figure 3), with trees and parameters sampled every 1,000 generations. The first one-fourth of all generations was discarded as a burn-in. The majority rule consensus tree of all remaining trees was calculated. Branches were considered significantly supported if they received a maximum likelihood bootstrap value (BS) ≥70%, a maximum parsimony bootstrap value (BT) ≥70%, or Bayesian posterior probabilities (BPP) ≥0.95.

## Results

### Phylogenetic analyses

The ITS+nLSU dataset (Figure 1) included sequences from 45 fungal specimens representing 45 species. The dataset had an aligned length of 3,095 characters, of which 1,910 characters are constant, 353 are variable and parsimony uninformative, and 832 are parsimony informative. Maximum parsimony analysis yielded 45 equally parsimonious trees (TL = 3,984, CI = 0.4666, HI = 0.5334, RI = 0.3909, and RC = 0.1824). The best model was GTR+I+G [lset nst = 6, rates = invgamma; prset statefreqpr = dirichlet (1,1,1,1)]. Bayesian and ML analyses showed a topology similar to that of MP analysis with split frequencies equal to 0.009126 (BI), and the effective sample size (ESS) across the two runs is double that of the average ESS (avg ESS) = 250.5.

The ITS+nLSU rDNA gene regions (Figure 1) were based on 13 orders, namely, Agaricales Underw., Atheliales Jülich, Boletales E.J. Gilbert, Cantharellales Gäum., Corticiales K.H. Larss., Gloeophyllales Thorn, Gomphales Jülich, Hymenochaetales Oberw., Polyporales, Russulales, Thelephorales Corner ex Oberw., Trechisporales, and Xenasmatales, while *Xenasmatella* was separated from the other orders.

The nLSU-alone dataset (Figure 2) included sequences from 58 fungal specimens representing 58 species. The dataset had an aligned length of 1,343 characters, of which 726 characters are constant, 176 are variable and parsimony-uninformative, and 441 are parsimony-informative. Maximum parsimony analysis yielded 3 equally parsimonious trees (TL = 2,864, CI = 0.3209, HI = 0.6791, RI = 0.4476, and RC = 0.1436). The best model for the ITS dataset estimated and applied in the Bayesian analysis was GTR+I+G [lset nst = 6, rates = invgamma; prset statefreqpr = dirichlet (1,1,1,1)]. The Bayesian and ML analyses resulted in a topology similar to that of MP analysis with split frequencies equal to 0.009830 (BI), and the effective sample size (ESS) across the two runs is double that of the average ESS (avg ESS) = 402.

The nLSU regions (Figure 2) were based on 13 orders, namely, Agaricales, Atheliales, Boletales, Cantharellales, Corticiales, Gloeophyllales, Gomphales, Hymenochaetales, Polyporales, Russulales, Thelephorales, Trechisporales, and Xenasmatales, while *Xenasmatella* was separated from the other orders.

The ITS-alone dataset (Figure 3) included sequences from 26 fungal specimens representing 15 species belonging to *Xenasma* and *Xenasmatella*. The dataset had an aligned length of 598 characters, of which 267 characters are constant, 74 are variable and parsimony-uninformative, and 257 are parsimony-informative. Maximum parsimony analysis yielded 1 equally parsimonious tree (TL = 629, CI = 0.7329, HI = 0.2671, RI = 0.8301, and RC = 0.6084). The best model for the ITS dataset estimated and applied in the Bayesian analysis was GTR+I+G [lset nst = 6, rates = invgamma; prset statefreqpr = dirichlet (1,1,1,1)]. The Bayesian and ML analyses resulted in a topology similar to MP analysis with split frequencies equal to 0.007632 (BI), and the effective sample size (ESS) across the two runs is double that of the average ESS (avg ESS) = 300.5.

In the ITS sequence analysis (Figure 3), a previously undescribed species was grouped into *Xenasmatella* with a sister group to *X. vaga* (Fr.) Stalpers.

### Taxonomy

**Xenasmatales** K.Y. Luo and C.L. Zhao, **ord. nov**.
MycoBank no.: MB 842882
Type family: Xenasmataceae Oberw.

Basidiomata resupinate. Hyphal systems are monomitic, generative hyphae with clamp connections. Basidia pleural. Basidiospores are colorless.

**Xenasmataceae** Oberw., Sydowia 19(1–6): 25 (1966).
MycoBank no.: MB 81527
Type genus: *Xenasma* Donk

Basidiomata resupinate, ceraceous to geletinous. Hyphal systems are monomitic, generative hyphae with clamp connections. Basidia pleural usually with 4 sterigmata and a basal clamp connection. Basidiospores are colorless.

***Xenasma*** Donk, Fungus, Wageningen 27: 25 (1957).
MycoBank no.: MB 18755
Type species: *Xenasma rimicola* (P. Karst.) Donk.

Basidiomata resupinate, adnate, are ceraceous to gelatinous when fresh, membranaceous when dry, and have a hymenophore smooth. Hyphal system are monomitic, generative hyphae with clamp connections. Cystidia and cystidioles are present. Basidia are cylindrical to subclavate, pleural, usually with 4 sterigmata and a basal clamp connection. Basidiospores are globose to cylindrical, colorless, thin-walled, warted to striate, non-amyloid, and weakly dextrinoid.

***Xenosperma*** Oberw., Sydowia 19(1–6): 45 (1966).
MycoBank no.: MB 18759
Type species: *Xenosperma ludibundum* (D.P. Rogers and Liberta) Oberw.

Basidiomata resupinate, closely adnate to the substratum, are gelatinous when fresh and pruinose when dry. Hyphal systems are monomitic, generative hyphae with clamp connections. Cystidia are absent. Basidia pleural, usually with 2–4 sterigmata and a basal clamp connection. Basidiospores are angular, colorless, thin-walled, tetrahedral, with some protuberances, IKI–, and CB–.

***Xenasmatella*** Oberw., Sydowia 19(1–6): 28 (1966).
MycoBank no.: MB 18756
Type species: *Xenasmatella subflavidogrisea* (Litsch.) Oberw. ex Jülich.

Basidiomata resupinate with a gelatinous. Hyphal system with clamped generative hyphae. Cystidia are absent. Basidia pleural, usually with 4 sterigmata and a basal clamp connection. Basidiospores are hyaline, thin-walled, warted, IKI–, and CB–.

**Figure 4:**
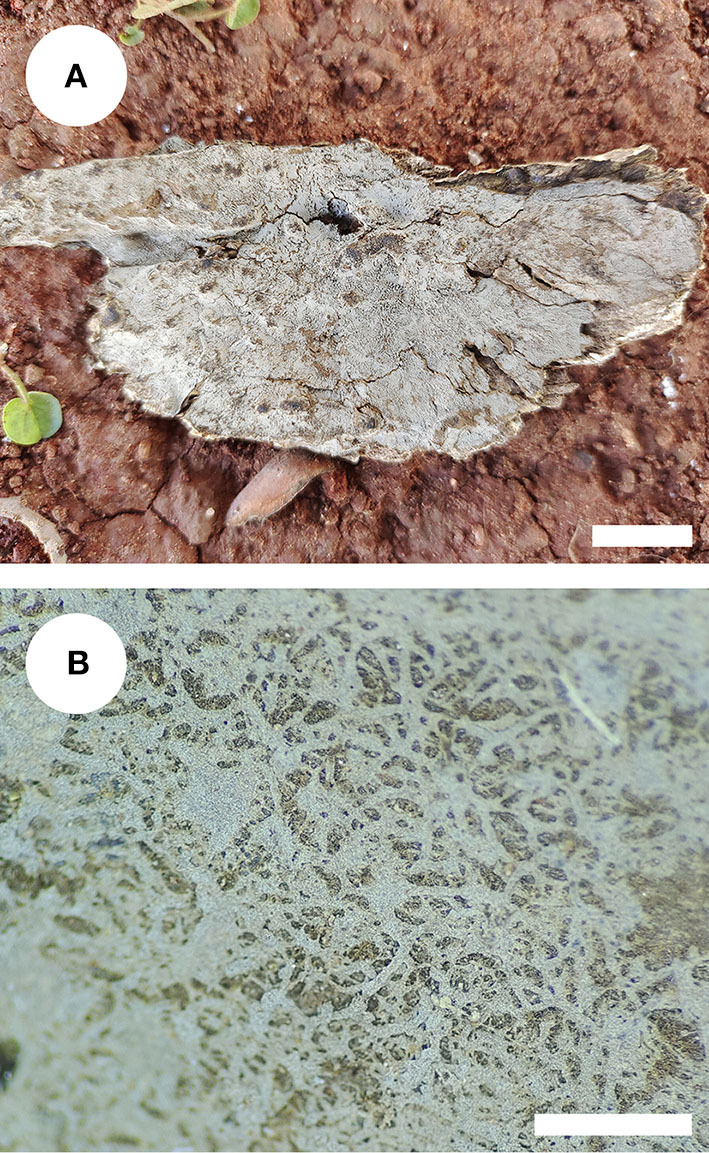
Basidiomata of *Xenasmatella nigroidea* (holotype). Bars: **(A)** 1 cm; **(B)** 1 mm.

**Figure 5:**
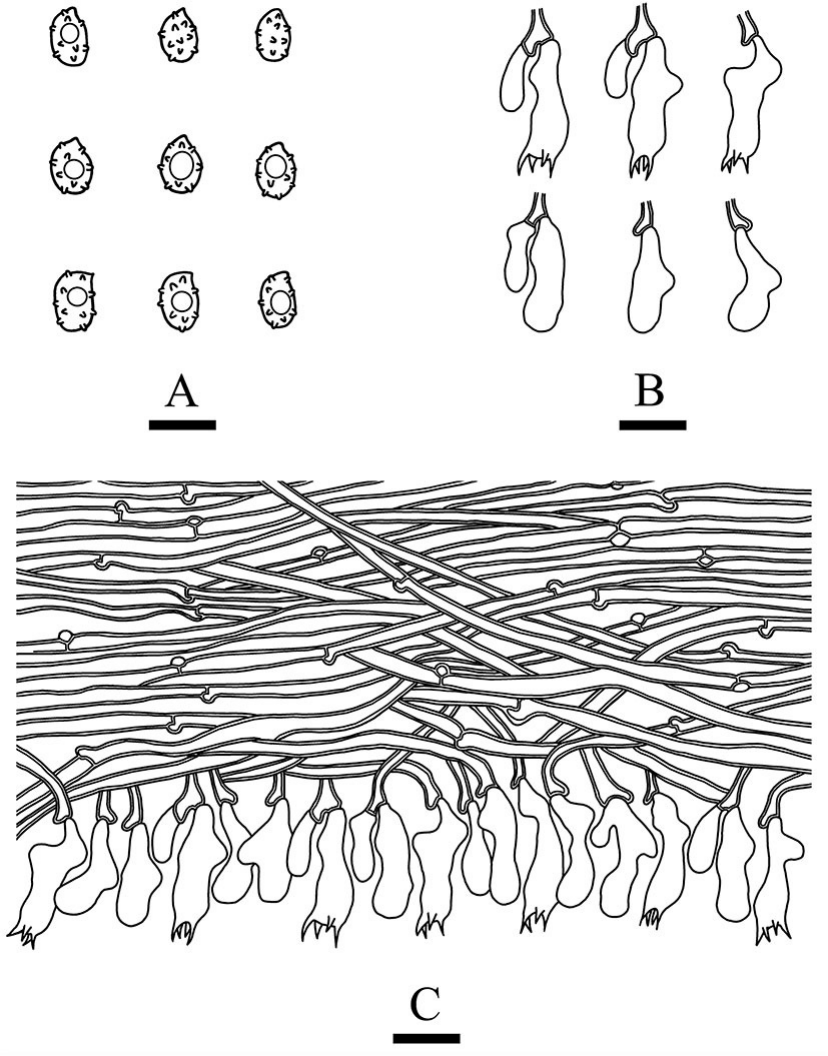
Microscopic structures of *Xenasmatella nigroidea* (drawn from the holotype). **(A)** Basidiospores. **(B)** Basidia and basidioles. **(C)** A section of hymenium. Bars: **(A)** 5 μm; **(B,C)** 10 μm.

***Xenasmatella nigroidea*** K.Y. Luo and C.L. Zhao, **sp. nov**.
*MycoBank no*.: MB 842470, Figures 4, 5.

Holotype—China. Yunnan Province, Honghe, Pingbian County, Daweishan National Nature Reserve, GPS coordinates 23°42′ N, 103°32′ E, altitude 1,500 m asl., on angiosperm stump, leg. C.L. Zhao, August 3, 2019, CLZhao 18333 (SWFC).

Etymology—*nigroidea* (Lat.): refers to the black hymenial surface.

*Basidiomata*: Basidiomata are annuals, resupinate, thin, very hard to separate from substrate, odorless or tasteless when fresh, grayish when fresh, gray to black and brittle when dry, up to 7.5 cm long, 3.5 cm wide, 70–150 μm thick. Hymenial is surface smooth, and byssaceous to reticulate under the lens. Sterile margin indistinct, black, up to 1 mm wide.

*Hyphal system*: monomitic, generative hyphae with clamp connections, thick-walled, unbranched, 2.5–4 μm in diameter, IKI–, CB–, and tissues unchanged in KOH.

*Hymenium*: cystidia and cystidioles are absent; basidia are pleural, clavate, with 4 sterigmata and a basal clamp connection, 12.0–18.0 × 4.5–6 μm; basidioles are shaped similar to basidia but slightly smaller.

*Basidiospores:* ellipsoid, colorless, thin-walled, warted throughout, asperulate with blunt spines up to 0.2 μm long, with one oil drop inside, IKI–, CB–, 3.5–4.5 × 2.5–3.5 μm, L = 4.07 μm, W = 2.87 μm, Q = 1.38–1.45 (*n* = 60/2).

*Type of rot*: White rot.

*Additional specimen examined*: CHINA, Yunnan Province, Honghe, Pingbian County, Daweishan National Nature Reserve, GPS coordinates 23°40′ N, 103°31′ E, altitude 1,500 m asl., on the angiosperm stump, leg. C.L. Zhao, August 3, 2019, CLZhao 18300 (SWFC).

## Discussion

There have been debates among mycologists regarding the order level taxonomic status of the Xenasmataceae. Corticioid homobasidiomycetes have a high phylogenetic diversity. Thus, an accurate place for the taxa of Xenasmataceae has not been decided. However, it was only assigned to euagarics clade (Larsson et al., 2004). Later, the Phlebiella family was proposed by Larsson (2007) on the basis of corticioid fungi; however, this group was not placed under any order. Recently, Xenasmataceae was placed under Russulales by He et al. (2019). Zong et al. (2021) studied the specimens and sequences from China and treated this group as *Xenasmatella* as the phylogenetic datasets showed that this clade does not belong to any order. In the present study (Figure 1), the ITS+nLSU analyses of 13 orders, namely, Agaricales, Atheliales, Boletales, Cantharellales, Corticiales, Gloeophyllales, Gomphales, Hymenochaetales, Polyporales, Russulales, Thelephorales, Trechisporales, and Xenasmatales showed that the taxa of Xenasmataceae form a single lineage with the sequences of Hymenochaetales and Atheliales; and this is similar to the results of Larsson (2007). In the present study (Figure 2), the nLSU analysis showed that the taxa of Xenasmataceae form a single lineage with the sequences of Hymenochaetales and Boletales; and this is similar to the results of Larsson (2007). In the present study (Table 2), we have enumerated morphological differences among the related orders. Therefore, a new fungal order, Xenasmatales, is proposed on the basis of morphological and molecular identification.

**Table 2:** Morphological characteristics of the relevant orders used in this study.

**Order Name**	**Morphological characteristics**	**References**
Agaricales	Hymenophore type gilled, poroid, ridged, veined, spinose, papillate, and smooth; spore deposit color white, pink, brown, purple-brown and black	Fries, 1821–1832, 1828, 1857–1863, 1874
Atheliales	Generally corticioid and athelioid, producing effused, crust like fruiting bodies that are loosely attached to the substrate and with non-differentiated margins	Eriksson et al., 1978, 1981, 1984
Boletales	Includes conspicuous stipitate-pileate forms that mainly have tubular and sometimes lamellate hymenophores or intermediates that show transitions between the two types of hymenophores. Also includes gasteromycetes (puffball-like forms), resupinate or crust-like fungi that produce smooth, merulioid (wrinkled to warted), or hydnoid (toothed) hymenophores, and a single polypore-like species, *Bondarcevomyces taxi*	Gilbert, 1931; Besl and Bresinsky, 1997; Jarosch, 2001; Larsson et al., 2004
Corticiales	Basidiomata resupinata, effuso-reflexa vel discoidea; hymenophora laevia; systema hypharum monomiticum; dendrohyphidia raro absentia; basidia saepe e probasidiis oriuntur. Cystidia presentia vel absentia. Sporae hyalinae, tenuitunicatae, albae vel aggregatae roseae.	Hibbett et al., 2007
Gloeophyllales	Basidiomata annua vel perennia, resupinata, effuso-reflexa, dimidiata vel pileata; hymenophora laevia, merulioidea, odontioidea vel poroidea. Systema hypharum monomiticum, dimiticum vel trimiticum. Hyphae generativae fibulatae vel efibulatae. Leptocystidia ex trama in hymenium projecta, hyalina vel brunnea, tenuitunicata vel crassitunicata. Basidiosporae laeves, hyalinae, tenuitunicatae, ellipsoideae vel cylindricae vel allantoideae, inamyloideae. Lignum decompositum brunneum vel album.	Hibbett et al., 2007
Gomphales	Basidiomata can be coralloid, unipileate or merismatoid (having a pileus divided into many smaller pilei); the pileus, if present, can be fan- to funnel-shaped	Gonzalez-Avila et al., 2017
Hymenochaetales	Hymenial structure (corticioid, hydnoid or poroid) and basidiocarps (resupinate, pileate or stipitate); the main characters are the xanthochroic reaction, the lack of clamps, the frequent occurrence of setae	Tobias and Michael, 2002
Thelephorales	Basidiospores tuberosae spinosaeque plus minusve coloratae	Oberwinkler, 1975
Trechisporales	Basidiomata resupinata, stipitata vel clavarioidea. Hymenophora laevia, grandinioidea, hydnoidea vel poroidea. Systema hypharum monomiticum vel dimiticum. Hyphae fibulatae, septa hypharum interdum inflata (ampullata). Cystidia praesentia vel absentia. Basidia 4-6 sterigmata formantia. Sporae laeves vel ornatae. Species lignicolae vel terricolae.	Hibbett et al., 2007
Xenasmatales	Basidiomata resupinate. Hyphal system monomitic, generative hyphae with clamp connections. Basidia pleural. Basidiospores colorless.	Present study

*Phlebiella* was not deemed to be a legitimately published genus (Duhem, 2010), and transferring to *Xenasmatella* was proposed. Later, Larsson et al. (2020) studied corticioid fungi (Basidiomycota and Agaricomycetes) and agreed with Duhem (2010), who suggested accepting the genus *Xenasmatella*. Recently, several mycologists have suggested the replacement of the invalid genus *Phlebiella* with *Xenasmatella* on the basis of morphology and molecular analyses (Maekawa, 2021; Zong et al., 2021).

On the basis of ITS dataset, a previous study showed that nine species of *Xenasmatella* have been reported, of which 6 new species were found in China, namely, *X. ailaoshanensis* C.L. Zhao ex C.L. Zhao and T.K. Zong, *X. gossypina, X. rhizomorpha, X. tenuis, X. wuliangshanensis*, and *X. xinpingensis*. According to our sequence data, *Xenasmatella nigroidea* was nested into *Xenasmatella* with strong statistical support (Figure 3), and formed a sister group with *X. vaga*. However, *X. nigroidea* is morphologically distinguished from *X. vaga* by larger basidiospores (5–5.5 × 4–4.5 μm). In addition, it turns dark red or purplish with KOH (Bernicchia and Gorjón, 2010).

Morphological comparisons of *Xenasmatella nigroidea* and other species are included in Table 3. *Xenasmatella nigroidea* is similar to *X. christiansenii* (Parmasto) Stalpers, *X. fibrillosa* (Hallenb.) Stalpers, *X. gossypina*, and *X. rhizomorpha* C.L. Zhao by having gossypine, byssaceous to reticulate hymenial surface, however, *X. christiansenii* is distinguished from *X. nigroidea* by its larger basidiospores (6–7 × 4–4.5 μm) and asperulate with blunt spines (up to 1 μm long; Bernicchia and Gorjón, 2010). *Xenasmatella fibrillosa* differs from *X. nigroidea* due to the presence of a white to pale yellowish white hymenial surface and longer basidiospores (4.5–5.5 μm; Bernicchia and Gorjón, 2010). *Xenasmatella gossypina* can be distinguished from *X. nigroidea* because it has cotton to flocculent basidiomata with a cream to buff hymenial surface and subglobose to globose basidiospores (Zong and Zhao, 2021). *Xenasmatella rhizomorpha* is separated from *X. nigroidea* by the clay-buff to cinnamon hymenial surface and the presence of the rhizomorphs (Zong et al., 2021).

**Table 3:** Morphological characteristic comparison of *Xenasmatella nigroidea* and other species.

**Species name**	**Basidiomata**	**Hymenial surface**	**Basidia**	**Basidiospores**	**References**
*Xenasmatella nigroidea*	Thin, very hard to separate from substrate	Smooth, byssaceous to reticulate under the lens	12–18 × 4.5–6 μm	Ellipsoid, 3.5–4.5 × 2.5–3.5 μm; asperulate with blunt spines up to 0.2 μm long	Present study
*X. christiansenii*	Fragile	Smooth, pruinose to farinaceous or more or less reticulate	6–7 × 4–4.5 μm	Ellipsoid, 6–7 × 4–4.5 μm; asperulate with blunt spines up to 1 μm long	Bernicchia and Gorjón, 2010
*X. fibrillosa*	Thin, fragile	Porulose to reticulate or formed by radially arranged, white to pale yellowish white	12–15 × 4–5 μm	Ellipsoid, 4.5–5.5 × 3–3.5 μm	Bernicchia and Gorjón, 2010
*X. gaspesica*	Small spots and becoming a closed coating, firmly attached	Resh smooth and somewhat gelatinous, light gray, dry waxy, white gray	7–11 × 4–4.5 μm	Ellipsoid, 8–10 × 2–2.5 μm	Grosse-Brauckmann and Kummer, 2004
*X. gossypina*	Cotton to flocculent	Cream to buff	14–23.5 × 4–7 μm	Subglobose to globose, 3.3–4.4 × 2.8–4 μm	Zong and Zhao, 2021
*X. odontioidea*	Colliculosa	Ceraceo-membranacea	17.5–20 × 4.5–5 μm	Ovale-ellipsoid, 2.5–3.5 μm	Ryvarden and Liberta, 1978
*X. rhizomorpha*	Presence of the rhizomorph	Clay-buff to cinnamon	10.5–17.5 × 3.5–6.5 μm	Ellipsoid, 3.1–4.9 × 2.3–3.3 μm	Zong et al., 2021
*X. subflavidogrisea*	Thin	White to grayish	10–12 × 4–5 μm	Ellipsoid, 3.5–4.5 × 2–2.5 μm	Bernicchia and Gorjón, 2010
*X. vaga*	Detachable	Grandinioid	15–20 × 5–6 μm	Ellipsoid, 5–5.5 × 4–4.5 μm	Bernicchia and Gorjón, 2010

*Xenasmatella nigroidea* is similar to *X. gaspesica* (Liberta) Hjortstam, *X. odontioidea* Ryvarden & Liberta, *X. subflavidogrisea* (Litsch.) Oberw. ex Jülich, and *X. vaga* (Fr.) Stalpers due to the presence of the ellipsoid or narrowly ellipsoid basidiospores. However, *X. gaspesica* differs from *X. nigroidea* because it has smaller basidia (7–11 × 4–4.5 μm) and larger basidiospores (8–10 × 2–2.5 μm; Grosse-Brauckmann and Kummer, 2004). *Xenasmatella odontioidea* can be distinguished from *X. nigroidea* by its colliculosa hymenial surface and shorter basidiospores (2.5–3.5 μm; Ryvarden and Liberta, 1978). *Xenasmatella subflavidogrisea* is separated from *X. nigroidea* due to the presence of a white to grayish hymenial surface, turning dark reddish brown in KOH and narrower basidiospores (2–2.5 μm; Bernicchia and Gorjón, 2010). *Xenasmatella vaga* differs from *X. nigroidea* due to its grandinioid hymenial surface and larger basidiospores (5–5.5 × 4–4.5 μm; Bernicchia and Gorjón, 2010).

**Figure 6:**
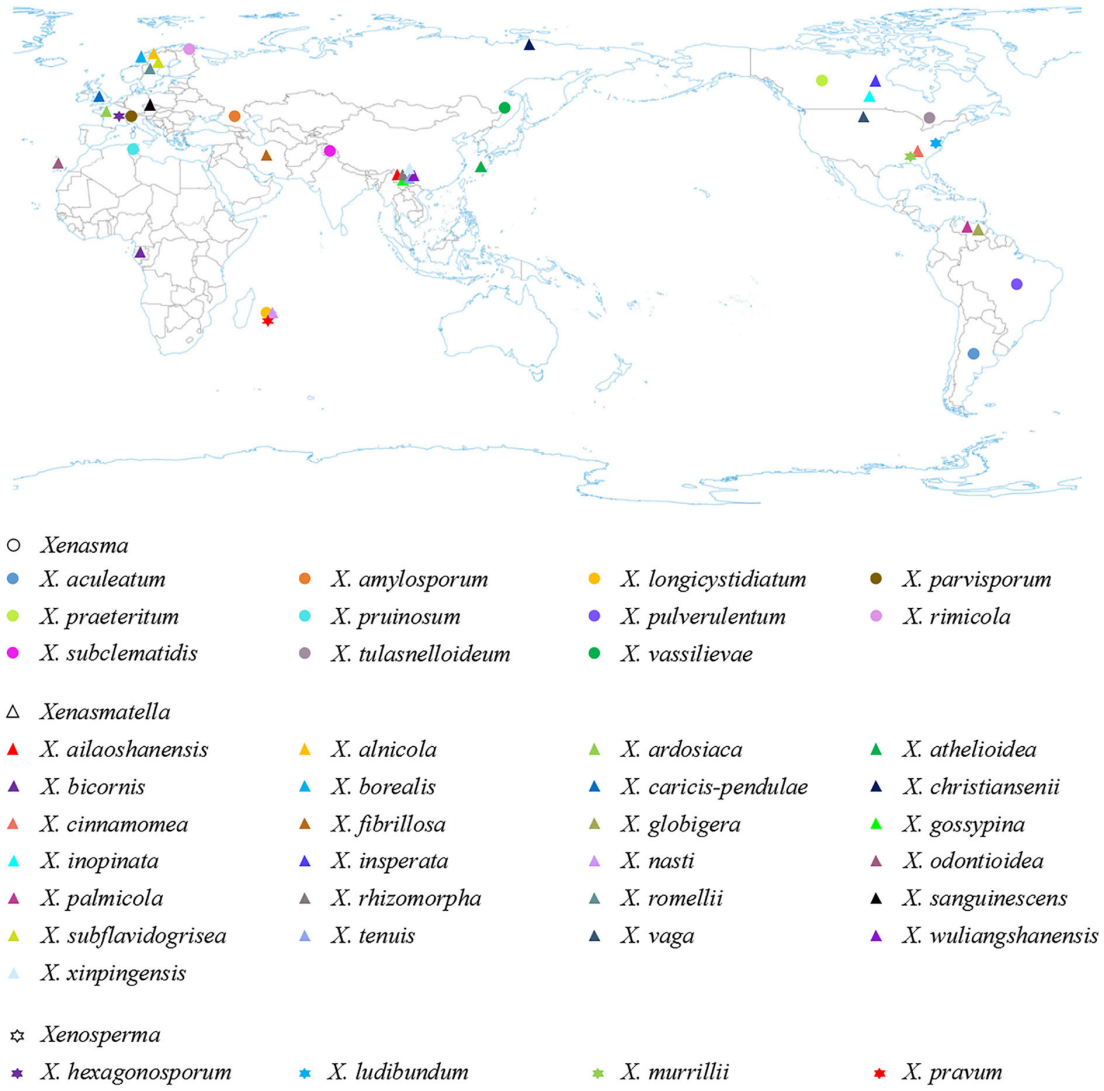
The geographic distribution of Xenasmataceae species (holotype) worldwide.

Based on the geographical distribution in America, Asia, and Europe, and ecological habits, white-rot causing Xenasmataceae have been reported in angiosperms and gymnosperms (Figure 6 and Table 4) (Stalpers, 1996; Dai et al., 2004; Hjortstam and Ryvarden, 2005; Bernicchia and Gorjón, 2010; Duhem, 2010; Dai, 2011; Huang et al., 2019; Larsson et al., 2020; Maekawa, 2021; Zong and Zhao, 2021; Zong et al., 2021). Key to 25 accepted species of *Xenasmatella* worldwide in Table 5. Many wood-decaying fungi have been recently reported worldwide (Zhu et al., 2019; Angelini et al., 2020; Gafforov et al., 2020; Zhao and Zhao, 2021). According to the results of our study on *Xenasmatella*, all these fungi can be classified into a new taxon (Figure 3). In addition, this study contributes to the knowledge of the fungal diversity in Asia.

**Table 4:** The geographic distribution and host-substratum of Xenasmataceae species (holotype).

**Species name**	**Geographic distribution**	**Host-substratum**	**References**
*Xenasma aculeatum*	Argentina	On fructifications of *Hypoxylon*	Gómez, 1972
*X. amylosporum*	Primorye	On rotten trunk of *Picea jezoensis*	Parmasto, 1968
*X. longicystidiatum*	Réunion	On *Rubus alcaefolius*	Boidin and Gilles, 2000
*X. parvisporum*	Czech Republic	On fallen branch of *Quercus petraea*	Pouzar, 1982
*X. praeteritum*	Ontario	On wood	Donk, 1957
*X. pruinosum*	Tunisia	On oak tree, bared and rotten	Donk, 1957
*X. pulverulentum*	Austria	On rotten wood	Donk, 1957
*X. rimicola*	Finland	On cracks in bark	Donk, 1957
*X. subclematidis*	Jammu-Kashmir	On log	Rattan, 1977
*X. tulasnelloideum*	America	On very rotten wood	Höhnel and Litschauer, 1908
*X. vassilievae*	Khabarovsk	On fallen trunk of *Taxus cuspidata*	Parmasto, 1965
*Xenasmatella ailaoshanensis*	Yunnan	On trunk of *Angiospermae*	Huang et al., 2019
*X. alnicola*	Allier	Sur bois humides, aune, saule blane	Bourdot and Galzin, 1928
*X. ardosiaca*	France	On decayed wood	Bourdot and Galzin, 1928
*X. athelioidea*	Japan	On rotten trunk of *Quercus*	Maekawa, 2021
*X. bicornis*	Gabon	Among shrubs on shore	Boidin and Gilles, 2004
*X. borealis*	Norway	On rotten *Pinus sylvestris*	Hjortstam and Larsson, 1987
*X. caricis-pendulae*	Great Britain	On dead attached leaf of *Carex pendula*	Roberts, 2007
*X. christiansenii*	Kamchatka	On fallen branch of *Larix kurilensis* var. *glabra*	Parmasto, 1965
*X. cinnamomea*	Florida	On *Magnolia*	Burdsall and Nakasone, 1981
*X. fibrillosa*	Iran	On decayed wood	Hallenberg, 1978
*X. globigera*	Venezuela	On hardwood	Hjortstam and Ryvarden, 2005
*X. gossypina*	Yunnan	On trunk of *Angiospermae*	Zong and Zhao, 2021
*X. inopinata*	Ontario	On *Tsuga canadensis*	Jackson, 1950
*X. insperata*	Ontario	On bark	Jackson, 1950
*X. nasti*	Reunion	Under Nastus borbonicus	Stalpers, 1996
*X. odontioidea*	Canary	On decayed wood	Ryvarden and Liberta, 1978
*X. palmicola*	Venezuela	On palm	Hjortstam and Ryvarden, 2007
*X. rhizomorpha*	Yunnan	On trunk of *Angiospermae*	Zong et al., 2021
*X. romellii*	Sweden	On deciduous wood	Hjortstam, 1983
*X. sanguinescens*	Czech Republic	On decayed wood	Svrcek, 1973
*X. subflavidogrisea*	Sweden	On rotten wood of *Pinus sylvestris*	Jülich, 1979
*X. tenuis*	Yunnan	On trunk of *Angiospermae*	Zong et al., 2021
*X. vaga*	Italy	On *Robinia pseudoacacia*	Stalpers, 1996
*X. wuliangshanensis*	Yunnan	On trunk of *Angiospermae*	Zong and Zhao, 2021
*X. xinpingensis*	Yunnan	On trunk of *Angiospermae*	Zong et al., 2021
*Xenosperma hexagonosporum*	France	On wood of *Platanus acerifolia*	Boidin and Gilles, 1989
*X. ludibundum*	Massachusetts	On bark of *Quercus* and decayed wood of *Chamaecyparis thyoides*	Jülich, 1979
*X. murrillii*	Florida	On branch of *Juniperus virginiana*	Gilbertson and Blackwell, 1987
*X. pravum*	Réunion	On dead branch	Boidin and Gilles, 1989

**Table 5:** Key to 25 accepted species of *Xenasmatella* worldwide.

1. Gloeocystidia present	*X. inopinata*
1. Cystidia absent	2
2. Basidia with 2, 3 sterigmata	*X. bicornis*
2. Basidia with 4 sterigmata	3
3. Basidia sterigmata > 5 μm in length	*X. nasti*
3. Basidia sterigmata < 5 μm in length	4
4. Basidiospores > 5 μm in length	5
4. Basidiospores < 5 μm in length	12
5. Basidiospores > 4 μm in width	6
5. Basidiospores < 4 μm in width	9
6. Basidiospores globose	*X. ardosiaca*
6. Basidiospores ellipsoid	7
7. Basidia < 6 μm in width	*X. vaga*
7. Basidia > 6 μm in width	8
8. Growth on dead angiosperm	*X. caricis-pendulae*
8. Growth on the trunk of gymnosperm	*X. christiansenii*
9. Basidiospores < 2 μm in width	*X. athelioidea*
9. Basidiospores > 2 μm in width	10
10. Hymenial margin with fimbriae	*X. romellii*
10. Hymenial margin without fimbriae	11
11. Hymenial surface arachnoid or byssoid	*X. borealis*
11. Hymenial surface smooth	*X. insperata*
12. Basidiospores subglobose to globose	13
12. Basidiospores ellipsoid to subcylindrical	17
13. Basidiospores thick-walled	*X. globigera*
13. Basidiospores thin-walled	14
14. Hymenial surface clay-pink to saffron	*X. wuliangshanensis*
14. Hymenial surface white to grayish or cream to buff	15
15. Generative hyphae thick-walled, unbranched	*X. xinpingensis*
15. Generative hyphae thin-walled, branched	16
16. Hymenial surface gossypine to byssaceous	*X. gossypina*
16. Hymenial surface pruinose to farinaceous	*X. ailaoshanensis*
17. Generative hyphae thick-walled	18
17. Generative hyphae thin-walled	19
18. Hymenial surface gray to black	*X. nigroidea*
18. Hymenial surface clay-buff to cinnamon	*X. rhizomorpha*
19. Growth on palm	*X. palmicola*
19. Growth on other plant	20
20. Growth on the bark of magnolia	*X. cinnamomea*
20. Growth on other wood	21
21. Basidiospores slightly thick-walled	*X. alnicola*
21. Basidiospores thin-walled	22
22. Basidia barrel-shaped	*X. tenuis*
22. Basidia cylindrical	23
23. Basidiomata ochreous	*X. odontioidea*
23. Basidiomata white to gray	24
24. Basidiospores > 3 μm in width	*X. fibrillosa*
24. Basidiospores < 3 μm in width	*X. subflavidogrisea*

## Data availability statement

The datasets presented in this study can be found in online repositories. The names of the repository/repositories and accession number(s) can be found in the article/supplementary material.

## Author contributions

C-LZ: conceptualization, resources, supervision, project administration, and funding acquisition. C-LZ and K-YL: methodology, software, validation, formal analysis, investigation, writing—original draft preparation, writing—review and editing, and visualization. Both authors have read and agreed to the published version of the manuscript.

## Funding

The research was supported by the National Natural Science Foundation of China (Project No. 32170004, U2102220) to C-LZ, the Yunnan Fundamental Research Project (Grant No. 202001AS070043) to C-LZ, the High-level Talents Program of Yunnan Province (YNQR-QNRC-2018-111) to C-LZ, and the Yunnan Key Laboratory of Plateau Wetland Conservation, Restoration, and Ecological Services (202105AG070002) to K-YL.

## Conflict of interest

The authors declare that the research was conducted in the absence of any commercial or financial relationships that could be construed as a potential conflict of interest.

## Publisher's note

All claims expressed in this article are solely those of the authors and do not necessarily represent those of their affiliated organizations, or those of the publisher, the editors and the reviewers. Any product that may be evaluated in this article, or claim that may be made by its manufacturer, is not guaranteed or endorsed by the publisher.
